# Predicting Addictive Vulnerability: Individual Differences in Initial Responding to a Drug’s Pharmacological Effects

**DOI:** 10.1371/journal.pone.0124740

**Published:** 2015-04-16

**Authors:** Douglas S. Ramsay, Salwa Al-Noori, Jason Shao, Brian G. Leroux, Stephen C. Woods, Karl J. Kaiyala

**Affiliations:** 1 Department of Oral Health Sciences, University of Washington, Seattle, WA, United States of America; 2 Department of Biostatistics, University of Washington, Seattle, WA, United States of America; 3 Department of Psychiatry and Behavioral Neuroscience, University of Cincinnati, Cincinnati, OH, United States of America; University of Granada, SPAIN

## Abstract

Considerable data suggest that individuals who appear minimally disrupted during an initial drug administration have elevated risk for abusing the drug later. A better understanding of this association could lead to more effective strategies for preventing and treating drug addiction. To investigate this phenomenon using a rigorous experimental model, we first administered the abused inhalant nitrous oxide (N_2_O) to rats in a total calorimetry and temperature system to identify groups that were sensitive or insensitive to the drug’s hypothermic effect. We then enrolled the two groups in a novel N_2_O self-administration paradigm. The initially insensitive rats self-administered significantly more N_2_O than sensitive rats, an important step in the transition to addiction. Continuous non-invasive measurement of core temperature and its underlying determinants during screening revealed that both groups had similarly increased heat loss during initial N_2_O administration, but that insensitive rats generated more heat and thereby remained relatively normothermic. Calorimetry testing conducted after self-administration revealed that whereas N_2_O’s effect on heat loss persisted comparably for both groups, initially insensitive rats actually over-responded by generating excess heat and becoming hyperthermic. Thus, rats with the greatest initial heat-producing compensatory response(s) appeared initially insensitive to N_2_O-induced hypothermia, subsequently self-administered more N_2_O, and developed hyperthermic overcompensation during N_2_O inhalation, consistent with increased abuse potential and an allostatic model of addictive vulnerability.

## Introduction

Although many people use drugs recreationally, only a minority develop a substance use disorder [1]. Marked variability in addictive vulnerability has spurred strong interest in identifying predisposing factors, with the goal of developing interventions to avert abuse later in life. More than 30 years ago, Schuckit and colleagues [2, 3] initiated an influential line of research that involved administering an alcohol challenge to young men with and without a family history of alcoholism. Numerous parameters were assessed such as body sway and subjective feelings of intoxication [4]. When the subjects were subsequently followed longitudinally, a “low level of response” to alcohol during the initial challenge emerged as the major predictor of future alcohol abuse [5, 6]. Considerable evidence now confirms that appearing to be relatively unaffected by an initial alcohol administration based on subjective measures of intoxication [7], as well as of various physiological and endocrine outcomes [3, 8], is indeed predictive of future alcohol use disorders. What is not known is *how* exhibiting little measured change to an initial drug administration predisposes to its abuse [9, 10].

A widely accepted hypothesis is that being relatively ‘insensitive’ to a drug promotes greater consumption as the individual strives to achieve a desired effect [2, 3] and that greater exposure to the drug over time promotes the development of greater addiction-related phenomena such as drug tolerance, withdrawal and dependence. An alternative perspective is that the key to understanding the association between the magnitude of measured changes during an initial drug challenge and subsequent drug use is to distinguish the pharmacological effects of the drug from the centrally mediated regulatory counter-response(s) an individual generates when confronted with these effects [11, 12]. This view prompted the hypothesis and its subsequent confirmation [13] that appearing initially insensitive to a drug can result from an individual making robust regulatory responses that compensate for a drug’s pharmacological effects, thereby minimizing the change in the measured variable. Accordingly, we hypothesized that an individual’s increased capacity for regulatory responding, rather than a true insensitivity to the drug’s pharmacological action, explains the association between appearing minimally impacted during an initial drug administration and having increased addictive vulnerability.

To investigate the latter hypothesis, we developed an experimental model that continuously and non-invasively measures the factors that determine an individual rat’s initial sensitivity to nitrous oxide (N_2_O)-induced hypothermia, and then assessed subsequent N_2_O self-administration in groups selected for high versus low initial hypothermia. In addition to assessing core body temperature (Tc) telemetrically, this paradigm allows simultaneous measurement of Tc’s underlying determinants, heat production (HP) and heat loss (HL), as assessed via combined direct and indirect calorimetry [14]. Previous research [13] has demonstrated that changes in HP during an initial N_2_O administration are the major determinant of individual differences in initial sensitivity to N_2_O -induced hypothermia. Thus, selecting individuals based on the magnitude of initial hypothermia primarily reflects underlying individual differences in HP responses that oppose the drug’s consistent pharmacological effect to increase heat dissipation [13]. This dissection of the underlying processes responsible for changes in Tc that occur during an initial N_2_O administration can then be compared with that same individual’s subsequent N_2_O self-administration behavior for possible associations.

N_2_O offers important advantages over other drugs of abuse for the purpose of this study. Its low solubility in blood and tissues enables a steady-state concentration to be quickly achieved and easily maintained such that intrasessional changes in the value of a measured variable cannot be attributed to changes in the drug’s pharmacological effects due to varying concentrations over time such as occurs on the ascending and descending limbs of a typical drug concentration curve [11]. Additionally, the lack of significant metabolic pathways for N_2_O [15] limits pharmacokinetic factors from influencing the drug’s pharmacological effect during the initial drug challenge. Finally, N_2_O is an abused inhalant drug [16] and, like other drugs of abuse, N_2_O supports self-administration in animals [17–19], and it has positively reinforcing (rewarding) effects for humans [20].

## Materials and Methods

### Experimental Overview

The experimental strategy partitioned the study into three phases. In Phase 1, rats were given an initial exposure to 60% N_2_O (challenge test) using total calorimetry so that those with the largest and smallest changes of Tc could be identified for further study. In Phase 2, these selected rats were then given the opportunity to self-administer N_2_O. In Phase 3, these same rats were tested again with a second challenge with 60% N_2_O in the total calorimetry apparatus.

### Subjects

Each squad of adolescent male, non-sibling, Long-Evans rats (Charles River, n = 189; eight squads of 23–24 rats per squad) arrived in the lab and the rats were housed in a polycarbonate tub (2–3 rats per tub) with free access to water and pelleted chow (5053 PicoLab Rodent Diet 20, Animal Specialties and Provisions, Quakertown, PA). The housing room and self-administration apparatus had a 12-h:12-h light/dark cycle (lights on at 0700 h). The lab’s ambient temperature was ~22 ± 1°C. On the day that each rat was given a 60% N_2_O challenge test to screen for individual differences in degree of initial sensitivity (9–14 d after arriving in the lab), the mean (SD) weight of the 189 screened rats was 160.2 ± 22.0 g.

### Ethics Statement

All animal procedures were approved by the University of Washington Institutional Animal Care and Use Committee. The Public Health Service assurance number issued by the Office of Laboratory Animal Welfare to the University of Washington is A3464-01.

### Total Calorimetry, Core Temperature (Tc), and N_2_O Administration Chambers

Six independent total calorimetry chambers that also measure Tc telemetrically served as gas exposure chambers. Total calorimetry simultaneously measures the two underlying determinants of Tc, the rates of total heat loss (HL) and metabolic heat production (HP). Dry HL was measured using a gradient layer direct calorimeter (Seebeck gradient layer calorimeter, SEC-A-0701, Thermonetics Corporation, La Jolla, CA; internal dimensions are 19 x 19 x 19 cm) that generates a millivolt signal directly proportional to the heat (sum of conduction, convection, and radiation) flowing across its gradient layer. Evaporative HL was calculated from the latent heat of vaporization of the water vapor added to the excurrent gas stream as previously described [14, 21]. Water vapor pressure was measured using a sensitive water vapor analyzer (model RH-300, Sable Systems, Las Vegas, NV). HP was calculated from oxygen consumption based on the modified Lusk equation as described elsewhere [14, 21]. Fractional oxygen concentrations in incurrent and excurrent gas streams were measured using the FoxBox oxygen analysis system (Sable Systems, Las Vegas, NV). Tc was measured telemetrically using a commercial system from Data Sciences International (Saint Paul, MN) that consists of a Data-Exchange Matrix, Physio-Tel Receiver (Model RPC-1), Dataquest ART 4.2 software, and an implantable battery-powered temperature sensor (model TA-F40) implanted in the rat’s peritoneal cavity. The antenna system within the direct calorimeter consists of two radio ferrite coils oriented perpendicularly to each other that are epoxied underneath a Plexiglas platform that holds them ~2 mm above the floor of the calorimeter. Wires from these coils exit the calorimeter through a sealed port and are connected to the commercial receiver base. All other instrument control and data acquisition were performed using custom programs written in LabVIEW 6.8 (National Instruments, Austin, Texas). A more detailed description of this system is available elsewhere [22].

Dependent variables obtained from the calorimetry tests were Tc, HP, dry HL and evaporative HL. Tc was recorded at 15-s intervals and mean Tc was calculated for each 6-min bin. HP and HL data were recorded at 10-s intervals. Average HP and HL were calculated for each 6-min bin. Gas concentration data were recorded from each calorimeter at 1-min intervals.

### N_2_O Self-administration Apparatus

Each 3-compartment self-administration apparatus consists of a standard rat housing tub open to room air that was customized with gas-tight side chambers protruding from its right and left sides. Four independent N_2_O self-administration chambers were arranged vertically (one per shelf) on a rolling cart. A photograph of the top two chambers is provided in S1 Fig. In brief, each apparatus was built by customizing a standard polycarbonate housing tub that had a wire grid floor insert elevated above the bedding material and a standard wire lid designed to hold rat chow and a water bottle. The central tub was modified so that a polycarbonate tube could be attached on each of its long sides, one on the right and one on the left. The tubes (diameter = 12 cm, length = 40 cm) have an internal volume of ~4.5 liters. A custom-machined collar was bonded to the outer-surface of the laterally positioned openings of the central tub and to both ends of each tube. These collars accommodated a rubber O-ring gasket. The side chambers were connected to the central tub by one of four types of “door” styles, i.e., 1) an open pass-through door, 2) a solid wall, 3) a freely swinging training door, or 4) a gas-tight, magnetically self-sealing, bi-directional door assembly. The end of each tube was closed with a nylon end-cap that had a clip mounted on its inner surface to hold a removable 50 ml liquid feeding tube (Bio-Serv, product #9019, Flemington, NJ) containing water. To determine when the rat was in a tube, five infrared LED beam detectors were positioned along the length of each side chamber with 8 cm spacing between detectors. The center LED was positioned at the middle of the tube length giving LED locations at 4, 12, 20, 28, and 36 cm from the entrance to the central tub.

### Gas Delivery to the Self-Administration Apparatus

A Parker Balston Lab Gas Generator (Model 74-5041NA) used room air to create a continuous supply of purified and dehumidified compressed air, which provided gas for the control gas condition. Compressed air from the lab gas generator was also used as a component of the gas blend to create the N_2_O gas condition.

A digital mass flow controller (Sierra Instruments Smart-Trak C50-AL-NR-2-PV2-V6-F3, range of 0–1.0 L/min) delivered 1.0 L/min of control gas to the side chambers designated to receive control gas. The 60% N_2_O gas condition was composed of 60% N_2_O, 21% oxygen, and 19% nitrogen which was made by blending medical grade oxygen, medical grade N_2_O, and control gas from the lab gas generator. Specifically, digital mass flow controllers blended 0.60 L/min of N_2_O (Smart-Trak C50-AL-NR-2-PV2-V6-SCR, range of 0–2.0L/min), 0.24 L/min of control gas (Smart-Trak C50-AL-NR-2-PV2-V6-SCR, range of 0–2.0L/min), and 0.16 L/min of oxygen (Smart-Trak C50-AL-NR-2-PV2-V6-SCR, range of 0–0.5 L/min) to deliver 1.0 L/min of 60% N_2_O to the side chamber assigned to receive N_2_O. Concentrations of N_2_O, oxygen, and carbon dioxide were measured using an infrared gas analyzer (Normocapoxy, Datex Instruments Corp., Helsinki, Finland) that drew gas samples via a t-connector placed in the excurrent gas line connected to each side chamber receiving N_2_O.

The time spent in each side chamber was calculated using infrared beam break data and the time remaining in a session was attributed to the central tub. Infrared beam breaks were scanned every 2 seconds in each side chamber and all of the beams broken during each 10 second interval were logged. The following algorithm was used to calculate how long a rat was inside a chamber. If any of the four beams furthest from the door were broken, that time interval was considered to have had the rat inside the chamber and the start of that 10-sec interval was considered the time of entry and the door entry count was incremented by one. Also, if only the single beam closest to the door was broken (during 1 or more consecutive 10-sec intervals), those interval(s) were scored as the rat being in chamber if and only if the next interval had a beam break in any of the four beams furthest from the door. Once the rat qualified as having entered the chamber, each subsequent 10-second sampling interval would be evaluated to determine whether the rat was still in the chamber. If any of the 5 beams were broken during that interval, the rat was considered to still be in the chamber. Should a 10-second interval have no beam breaks, it was considered indeterminate and would be classified based on the results of the next 10-sec interval. If no beam breaks occurred during the 2^nd^ consecutive 10-sec sampling interval, the rat was considered to have left the chamber and the exit time was logged as the end time of the last 10-second interval that did qualify as having the rat inside.

### Surgical placement of the telemetric temperature sensor

The telemetric temperature sensor was implanted surgically into each rat’s peritoneal cavity under isoflurane anesthesia while the rat was on a 39°C heating pad. Meloxicam (an NSAID) was provided in the drinking water (0.02 mg/ml H2O) from 1 d before to 2 d after surgery.

### Experimental Procedures

#### Overview

Rats are given an initial 60% N_2_O exposure (challenge test) using total calorimetry so that the most sensitive (initially sensitive, IS) and the least sensitive (initially insensitive, II) individuals to N_2_O’s hypothermic effect can be selected for further study. These rats are then allowed to self-administer N_2_O. Two days following the end of the chronic self-administration phase, these rats are assessed using total calorimetry in a final 60% N_2_O exposure session and control gas exposure session to measure the degree of tolerance / allostasis development. Following completion of this final assessment phase, subjects were euthanized using carbon dioxide inhalation.

#### Phase 1. Selecting for individual differences in initial sensitivity to 60% N_2_O

Within 6 d of arriving in the lab, each rat had a telemetric Tc sensor placed intraperitoneally. After at least 1 wk of post-surgical recovery, each rat was individually administered 60% N_2_O using the total calorimetry system. The testing procedure began with a 2-h (1000–1200 h) baseline administration of control air (custom blended air) delivered at a flow rate of 1.5 LPM. At 1200 h, a 1.5 LPM flow rate of N_2_O commenced to quickly establish and maintain a 60% N_2_O concentration for 3 h as described elsewhere [22]. At 1500 h, control gas was again delivered at 1.5 LPM for 45 min after which the rats were returned to their home cage. Using each rat’s Tc data, z-scores were calculated for individual rats in that squad and the 2 most sensitive (initially sensitive, IS) and the 2 least sensitive (initially insensitive, II) to 60% N_2_O’s effect on Tc were selected to proceed to the next phase of the study [23, 24]. The weight of the rats selected for the IS (n = 16) and II (n = 16) groups on the morning of the initial 60% N_2_O administration were: IS group = 153.9 ± 21.2 g; II group = 162.1 ± 23.9 g.

#### Phase 2. Self-administration of N_2_O

Approximately 16 d after arriving in the lab and at least 2 d following the initial N_2_O screening exposure, the four selected rats from each squad began the self-administration procedures which consisted of 4 parts: 1) training to enter side chambers, 2) training with magnetic doors, 3) N_2_O self-administration with water only available in both side chambers, 4) N_2_O self-administration with water only available in the central tub. Throughout the self-administration study, gas (control air or 60% N_2_O) was continuously delivered at a flow rate of 1 LPM to each side chamber and rats were placed in a holding tub between 1000–1100 h each day while the self-administration apparatus was serviced. Rats were also provided water between 1000–1100 h each day if they had not accessed water during the previous 23 hours. Rats lived in the self-administration apparatus for 29 consecutive days.

#### Training to enter side chambers

At 1100 h on Day 1, each rat was placed in the central tub of its assigned self-administration apparatus. An entirely open door was connected to one side chamber while entry to the other side chamber was prevented using a completely sealed door. Rat chow was available in the central tub and water was available at the end of the accessible side chamber. At 1000 h the next day, the rat was placed in a holding tub while the self-administration apparatus was serviced. At 1100 h on Day 2, this procedure was repeated with the open and closed doors reversed for the two side chambers. At 1100 h on Day 3, this procedure was repeated again except with the modification that the entirely open door was replaced with a freely swinging training door. The training door swung easily in both directions and was designed with a 1.75-cm gap at the bottom to encourage entry into the side chamber. At 1100 h on Day 4, this procedure was repeated with the training door and closed door reversed for the side chambers. Thus, this training procedure was conducted over 4 consecutive d and the door type connected to each side chamber was counterbalanced by side and group assignment across squads. Several times a day, rats were manually guided by the lab technician to enter the accessible side chamber.

#### Training with magnetic doors

For the next consecutive 9 days (rats entered the central tub by 1100 h and were placed in the holding tub the next day between 1000–1100 h), rats were trained to go through a gas-tight self-sealing magnetic door to enter the right and left side chambers that both contained control gas. Water was only available in the side chambers. Several times a day, the lab technician manually guided the rats to enter both side chambers until the rats were using the doors independently. Thus, this training procedure took place from Days 5–13.

#### N_2_O self-administration with water only available in both side chambers

For 8 consecutive days, from 1100 h until 0900 h the next day, 60% N_2_O was delivered to one side chamber and control gas to the other side chamber. Water was only available at the end of both side chambers, which means that a rat must enter one or the other (or both) side chambers to access water. The side chamber that received N_2_O alternated daily, with the initial side assignment being randomly determined and counterbalanced among and within groups. Lab personnel only entered the self-administration room from 1000–1100 h each day to service the equipment. Between 0900–1000 h, control air was delivered to both side chambers to clear the side chamber (and room) from N_2_O before the lab personnel entered. This schedule provided the rats with 22 h of access to N_2_O each day. Importantly, the rats were not disturbed by research staff from 1100 h until 1000 h the next day. This N_2_O self-administration procedure took place from Days 14–21.

#### N_2_O self-administration with water available only in the central tub

For 8 consecutive days, the procedures were identical to the previous 8 days except that the water was removed from the side chambers and was now *only* available via a water bottle placed beside the food on the lid of the central tub. Thus, the rats no longer needed to enter a side chamber to access water. This N_2_O self-administration procedure took place from Days 22–29.

#### Phase 3. Final Total Calorimetry Test Sessions

Rats were returned to the colony room for 3 d at the end of the self-administration procedure. On the next 2 d, the rats were tested twice (once per day) in the total calorimeter using the same timing and procedures as during the initial screening test. The session that delivered 60% N_2_O was identical to the initial screening test using 60% N_2_O and the other session only differed because control air was delivered during the entire session. The order of the two final calorimetry test sessions was counterbalanced within group assignment and squad. The weights of the rats in the IS (n = 16) and II (n = 16) groups on the morning of the final calorimetry test session were: IS group = 360.9 ± 35.0 g; II group = 384.5 ± 41.8 g.

### Statistical Analyses for Compositional Self-Administration Data

The primary outcome variable for assessing N_2_O self-administration is the amount of time each rat spent in each of the three compartments of the self-administration apparatus (i.e., the side chamber containing 60% N_2_O, the control side chamber containing compressed air, and the central tub). For each rat, the time spent in each compartment type was summed over the two 22-h measurement periods of each consecutive pair of days (dyads), thereby equating the availability of N_2_O on both sides within a dyad and controlling for an individual’s possible preference for a specific side chamber. Dyads 1 through 4 represent the first four pairs of self-administration days (Days 1–2, 3–4, 5–6, and 7–8) when water was provided only in the side chambers. Dyads 5 through 8 represent the next four pairs of self-administration days (Days 9–10, 11–12, 13–14, and 15–16) when water was available only in the central tub.

The self-administration data consist of the individual times spent in each of the three chambers during 44-h test-session dyads. Because these values must sum to a fixed, known value for each dyad, the data are considered compositional. If standard methods are used to analyze the raw data, spurious correlations arise due to the restricted sum [25]. We emphasize that methods for analyzing compositional data analysis have a long history, are rigorously justified, well developed from a computational perspective, and widely applied in many fields [26, 27]. An established way to present compositional data is in ternary plots, in which time or percent time spent in each chamber is plotted on one of three axes forming an equilateral triangle. We represented the central tendency of values on the ternary plot using methods described by Aitchison [25].

An accepted way to analyze compositional data is to work with ratios between the observed values instead of the raw values themselves [28]. Thus, our primary outcome of interest in all inferential models was a preference ratio, calculated as the time spent by each rat in the N_2_O chamber divided by the time it spent in the control chamber in the same dyad. To address the primary hypothesis, a linear regression was performed with the logarithm of the preference ratio as a continuous outcome observed in two groups defined by initial categorization of N_2_O sensitivity, adjusting for dyad as a continuous variable. We used generalized estimating equations (GEE) with robust (sandwich) standard errors to account for within-subject correlation. The same analysis was performed separately for data from Dyads 1–4 and Dyads 5–8. We were interested primarily in whether the preference ratio is positively associated with initial N_2_O insensitivity group, averaging across Dyads. In a secondary analysis (also performed separately for Dyads 1–4 and 5–8) we added a dyad-by-group interaction, to determine whether the preference ratio in each group changed over time.

#### Missing data

Two II and two IS rats selected from the sixth squad were missing self-administration data during parts of Dyad 4 and Dyad 5. We proposed *a priori* to not include all dyads in which data were missing. To check the assumption that the missingness occurred at random, we performed sensitivity analyses by imputing minimum and maximum values for the missing time data and re-fitting primary inferential models. We verified that the procedure did not appreciably affect the results.

#### Zero time values

It is possible that a rat does not spend any time in a given chamber during a dyad. Thus, a zero value would occur if a rat avoided one or both side chambers completely for both 22-hour sessions in a dyad. Because zero values are problematic when taking log-ratios, we added 5 sec to all zero values, which equaled one-half the lower limit of detection (i.e., 10 sec). As discussed extensively by Schilling and colleagues [28], this assumes that zero values for side chambers do not indicate that a rat would have continued exclusively occupying the central chamber (known as a structural zero). We felt that this assumption was reasonable because the experimental design during the first four dyads encouraged rats to eventually enter side chambers to access water. To check the robustness of our models to the imputed value, we tried adding a variety of values lower than 10 seconds, none of which resulted in appreciably different inferential results.

## Results

The Results section reports the findings for each phase of the study and includes abbreviated methodological details to improve clarity. The data underlying these findings are provided in S1 Dataset.

### Phase 1: Initial Exposure to 60% N_2_O Using Total Calorimetry

**Fig 1:**
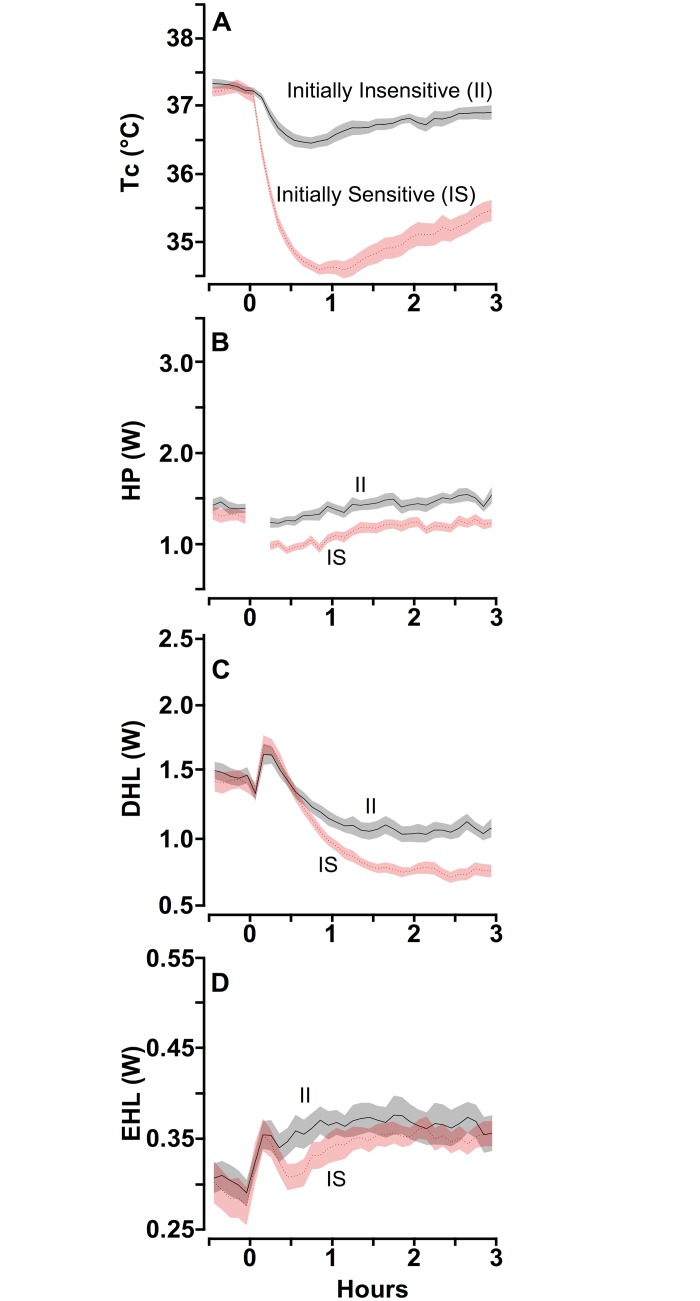
Calorimetric Assessment During an Initial 60% N_2_O Administration Distinguishes Initially Sensitive from Initially Insensitive Rats. Thermal profiles ± pointwise SE (shaded areas) of rats designated as initially sensitive (IS, n = 16) and initially insensitive (II, n = 16) based upon a N_2_O screening test of n = 189 candidates. A: Core temperature (Tc); B: heat production (HP); C: dry heat loss (DHL), and D: evaporative heat loss (EHL). HP data between 0 and 12 min are not depicted because it has been documented that the initiation of N_2_O delivery can cause a transient artifactual effect on HP [14].

Non-sibling adolescent male Long-Evans rats (n = 189) were exposed to 60% N_2_O for 3 h in a total calorimetry apparatus. Consistent with prior findings [23, 24], considerable inter-individual variability in the pattern of Tc was apparent. For each of the 8 squads screened (23–24 rats/squad), the 2 rats with the largest decrease of Tc and the 2 rats with the smallest decrease of Tc were selected for the self-administration phase of the study using previously described criteria [23, 24]. Therefore, the selection procedure yielded two groups of 16 rats each distinguished by the magnitude of the change in Tc during an initial exposure to 60% N_2_O (Fig 1A).

Rats with the greater change of Tc experienced a mean decrease of 2.66 ± 0.10°C after 70 min and had only partially recovered by the end of the third hour, remaining 1.79 ± 0.11°C below baseline. Thus, based on the change of Tc, these rats appear to be highly sensitive to N_2_O -induced hypothermia and in accord with customary usage are labeled IS (Initially Sensitive). In contrast, rats in the other group, although having a comparable baseline Tc as the IS rats, experienced a mean decrease of Tc of only 0.80 ± 0.10°C at 45 min, and this was followed by a gradual recovery toward baseline over the ensuing 2 h, remaining 0.35 ± 0.11°C below baseline. Thus, based on the relatively small change of Tc, rats in this group appear to be insensitive to N_2_O -induced hypothermia and are labeled II (Initially Insensitive). Dry heat loss (DHL) increased rapidly and similarly for the IS and II groups during the first 15 min of N_2_O (Fig 1C), and then decreased below basal levels, with a greater reduction observed for the IS than the II group in accordance with the smaller gradient between ambient temperature and Tc for the IS group compared to the II group. Evaporative heat loss (EHL; Fig 1D) increased for both groups after the onset of N_2_O and remained elevated throughout the 3-h N_2_O exposure, with the II group having greater EHL than the IS group for the duration of the N_2_O exposure. Despite the II group having a substantially smaller reduction in Tc than the IS group (Fig 1A), the II group actually had greater total heat loss (DHL plus EHL) than the IS group during N_2_O administration (means during 3-h N_2_O administration were 1.51 ± 0.22 (II) vs. 1.27 ± 0.16 W (IS) (p = 0.001) (Fig 1C and 1D). The important point is that Tc was less disrupted in the II group than in the IS group due to the II group’s significantly greater heat production (HP) response; averaged across the 3-h N_2_O exposure, HP was 1.41 ± 0.22 (II) vs. 1.14 ± 0.17 W (IS; p = 0.003) (Fig 1B). Moreover, in each of three hours of N_2_O administration, a greater number of the 16 II than 16 IS rats exhibited mean *increases* of HP from baseline [1^st^ h: 6 vs. 0 (p = 0.02 by 2-sided Fishers exact test); 2^nd^ h: 8 vs. 4 (p = 0.27); 3^rd^ h: 10 vs. 3 (p = 0.03)]. These findings support previous research indicating that inter-individual variability in the initial magnitude of N_2_O -induced hypothermia results primarily from individual differences in the amount of heat produced that opposes the N_2_O -induced increase in heat dissipation [13].

### Phase 2: Self-Administration of 60% N_2_O

All rats in both the II and IS groups then participated in the self-administration study. [A video clip of a rat using the self-administration apparatus is available online in S1 Movie]

**Fig 2:**
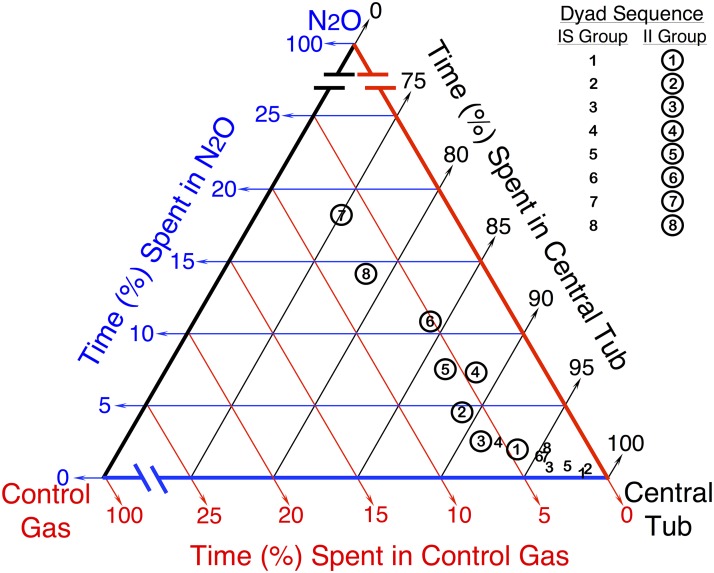
N_2_O Self-Administration Data for Initially Sensitive and Initially Insensitive Rats. Ternary diagram depicting the centered geometric mean proportions of time spent in each chamber for each of the eight 44-h dyads during N_2_O availability. The numbers 1–8 depicted in the figure represent the sequential order of self-administration dyads. Circled numbers represent dyads for initially insensitive rats (II, n = 16) while uncircled numbers represent dyads for the initially sensitive rats (IS, n = 16). The position of each point relative to the three color-coded axes indicates the proportion of time spent in the central tub (black), control gas chamber (red), and 60% N_2_O chamber (blue) during a 44-h dyad.

The primary outcome variable for assessing N_2_O self-administration is based on the amount of time each rat spent in each of the three compartments of the self-administration apparatus (i.e., the side chamber containing 60% N_2_O, the control side chamber containing compressed air, and the central tub). As explained previously, since the total proportions of time spent in all three compartments must sum to unity, these data are considered compositional [25]. A standard method for representing the central tendency of compositional data is to use a centered geometric mean, which is rescaled so that its components sum to unity [26]. The centered geometric mean proportions of time that the two groups spent in each of the three compartments during the N_2_O self-administration phase are plotted for each dyad on a ternary diagram (Fig 2). The ternary diagram depicts the overall pattern of results, with the II group steadily increasing the time spent in the N_2_O chamber over dyads and the IS group exhibiting negligible proclivity to self-administer N_2_O. [The times each individual rat spent in each compartment type during each dyad are provided in S2, S3 Figs.]

The dependent variable in all inferential models was a preference ratio based on the amount of time each rat spent in the side chamber containing N_2_O versus the control side chamber during a 44-hour dyad. Using a time ratio as the outcome removes dependency on the amount of time spent in the central tub, and is standard practice when considering compositional data [25, 28]. We fit linear models comparing the log-transformed preference ratio between II and IS rats, separately using data from Dyads 1 thru 4 (when water was available only in the side chambers) and Dyads 5 thru 8 (when water was available only in the central tub). In both models, we adjusted for dyad as a linear continuous variable and used GEE to account for within-subject correlation [29]. We also included an interaction term between dyad and group assignment.

**Fig 3:**
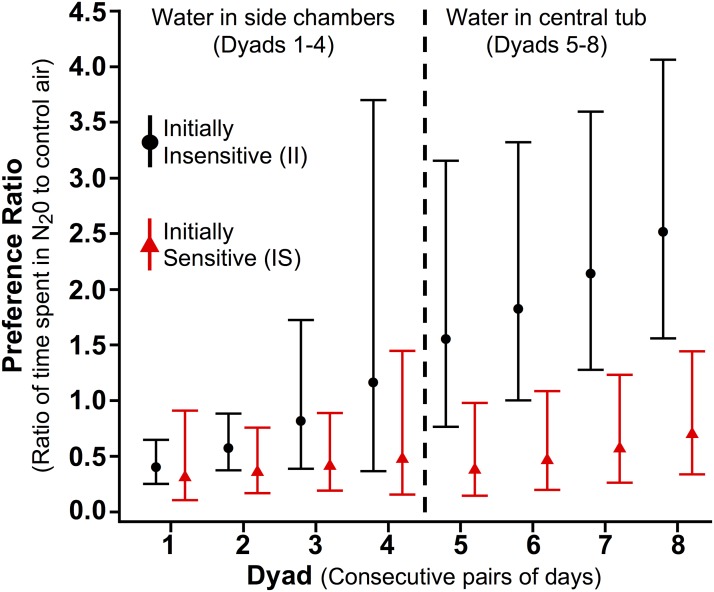
Statistical Comparison of the N_2_O Preference Ratio for Initially Sensitive and Initially Insensitive Rats. The N_2_O preference ratio is the ratio of time each rat spent in the N_2_O chamber versus the control air chamber during each dyad, and was compared between initially insensitive (II, black circles) and initially sensitive (IS, red triangles) rats using linear modeling applied to log-transformed ratios. Points represent geometric means and error bars are pointwise 95% confidence intervals. Ratio comparisons were statistically significant in each of Dyads 5–8. During Dyads 5–8, the II group’s mean N_2_O preference ratio was 3.68-fold higher than that of IS rats (95% CI: 1.48–9.20; p = 0.003). For Dyads 1–4, the preference ratio was 1.84 times greater than that of the IS group (95% CI: 0.80–4.40), but this difference did not reach statistical significance (p = 0.075). The group-by-dyad interactions were not statistically significant for Dyads 1–4 (p = 0.227) or for Dyads 5–8 (p = 0.343).

The results of the linear models are displayed in Fig 3. On average, the IS group spent less time in the N_2_O chamber than in the control chamber during all eight dyads, i.e., the mean ratios are all below 1:1. During Dyads 5 through 8, the ratio was significantly higher in II rats than in the IS ones, with II rats spending, on average, more than twice as much time in N_2_O versus the control chamber during the last two dyads (see Fig 3 legend for more details). The analysis also suggests that the difference between IS and II groups increased over dyads, although the interaction terms were not statistically significant.

### Phase 3: Final 60% N_2_O Exposure Using Total Calorimetry

Following completion of the self-administration phase, the rats were returned to the colony housing room for 3 d. Then, on each of the next 2 d, the rats were tested individually using combined direct and indirect calorimetry as during the initial N_2_O exposure. One test session delivered 60% N_2_O exactly as was done during the initial screening evaluation in Phase 1, and the other was identical except that only control gas was delivered. The order of the two sessions was counterbalanced within and between groups. Comparison of the initial and final N_2_O exposures effect on Tc (Figs 1A vs. 4A) reveals that the intervening N_2_O exposures that occurred during the self-administration phase engendered an adapted state in both the IS and II groups with features that depart from the standard definition of chronic tolerance wherein the measured outcome stays at or near baseline during drug administration in drug-adapted individuals.

**Fig 4:**
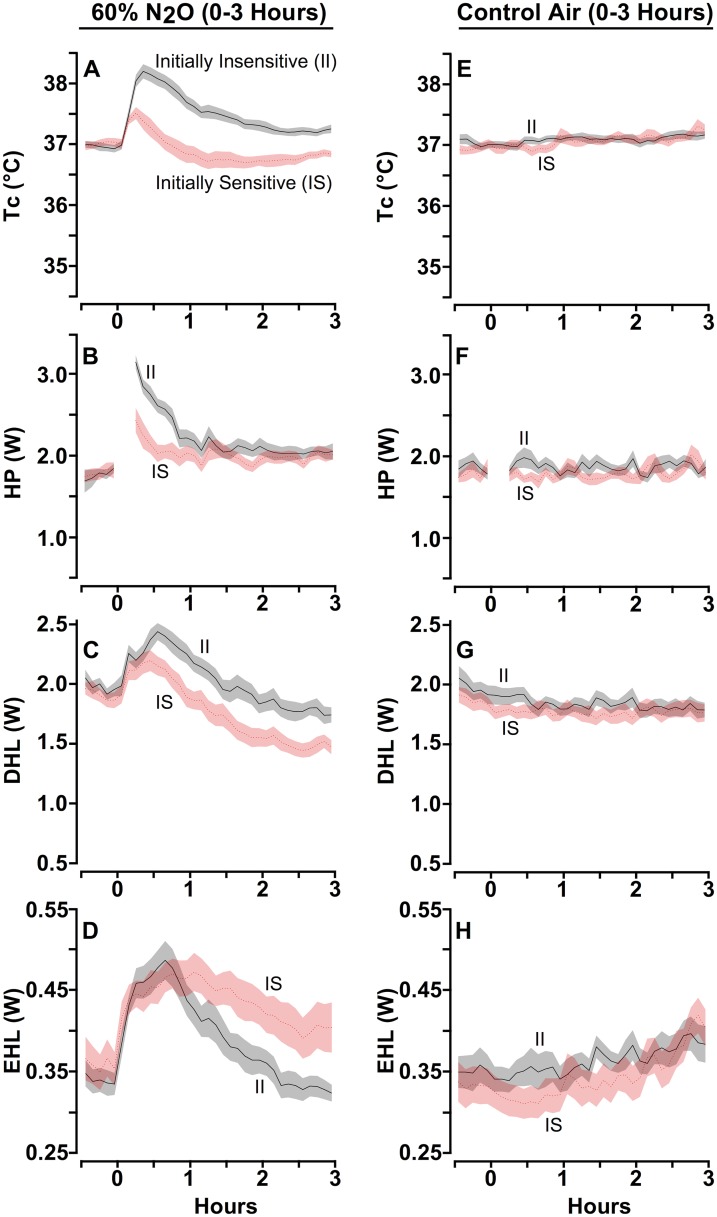
Final Calorimetric Assessment during a 60% N_2_O Administration for Initially Sensitive and Initially Insensitive Rats. A calorimetry retest conducted after the self-administration phase revealed that the IS and II groups both became frankly hyperthermic with the onset of 60% N_2_O administration but the magnitude was markedly greater and more persistent in the N_2_O self administration-prone II rats (A) due primarily to a greater HP response (B). The increases in DHL (C) and EHL (D) likely reflect a durable pharmacological effect of N_2_O in addition to the effect of increased body heat content to promote heat loss. The II and IS groups did not differ during the control gas session (E, F, G, H).

Specifically, both groups exhibited a brisk increase of Tc upon commencement of N_2_O administration (Fig 4A). In the IS group this hyperthermia peaked at 0.47 ± 0.13°C above baseline at 15 min into N_2_O administration but then resolved and subsequently decreased to 0.20 ± 0.07°C below baseline at the end of N_2_O administration such that the IS rats were hypothermic for the final 2 h. In contrast, Tc of the II rats peaked at over 1.25 ± 0.11°C above baseline and then also declined, but to a plateau that remained above baseline for the duration of the administration. Thus, while rats in both groups had similar dynamic profiles of Tc, the groups differed markedly in terms of magnitude; i.e., the II rats were frankly hyperthermic during the entire trial, and this outcome reflected a greater increase of HP (Fig 4B; change from baseline averaged across the N_2_O administration period and adjusted for baseline HP: II = 0.41 ± 0.056 W vs. IS = 0.20 ± 0.056 W; p = 0.011). Importantly, although the IS group’s acquired increase in HP was elevated throughout the final N_2_O exposure (Fig 4B), the magnitude and duration were only sufficient to transiently oppose N_2_O’s hypothermic effect in the face of total HL during the exposure session. In contrast, the rats categorized as initially insensitive did not merely blunt N_2_O’s hypothermic effect, but in fact *over*compensated for total HL and therefore sustained a *hyper*thermic state throughout the duration of the final N_2_O exposure.

The early session increases in DHL and EHL that occurred upon the commencement of N_2_O delivery during its initial administration (Fig 1C and 1D) recurred during the re-test (Fig 4C and 4D), implying that the heat loss effect of N_2_O (i.e., N_2_O’s pharmacological effect) persisted despite repeated N_2_O exposures. Importantly, the heat production responses changed markedly across the self-administration period, and these changes differed between the two groups. Hypothermic tolerance and hyper-tolerance developed primarily due to an augmented heat production response that countered the drug effect, as observed previously [30]. It is notable that the II group’s greater propensity for self administering N_2_O is associated with that group’s more robust *hypo*thermia-opposing HP response in the initial calorimetry test and its augmented *hyper*thermia-promoting HP response in the follow-up calorimetry test. Accordingly, an initially robust HP response appears to be a unifying mechanism for an addiction-related phenotypic triad consisting of a minimal change of Tc during an initial drug challenge, rapid development of chronic drug tolerance followed by a greater hyperthermic sign reversal, and an increased propensity for self-administering N_2_O.

## Discussion

Rats that generated a more robust compensatory response when initially challenged by N_2_O were significantly and substantially more likely to self-administer that drug when given the opportunity. These findings are consistent with, and add importantly to, the reports by Schuckit [3, 8] and others [7] that individuals exhibiting the smallest change in a measured parameter during an initial drug challenge are more likely to self-administer the drug in the future. A critical distinction, however, is that the current results, as well as previous studies [13, 30], demonstrate with clarity that it is inaccurate to characterize rats that appear initially insensitive to drug-induced hypothermia as being genuinely insensitive to the drug’s pharmacological effects. Indeed, in our study, N_2_O -induced increases in early HL are similar for both IS and II rats, and the overall increase in HL was actually greater in II than in IS rats. These groups differed at the level of Tc because they differentially activated heat-producing responses.

The capacity for reacting to a drug-induced disturbance (here, increased HL) with the activation of compensatory effector responses (here, increased HP) is an underappreciated mechanism for reducing the magnitude of change in a conveniently measured variable (here, Tc). We stress that the variables that have been traditionally employed to measure initial drug sensitivity represent choices of convenience that in fact integrate the impacts of many (usually unmeasured) pharmacological effects and regulatory responses [11, 12]. Traditional variables for drug sensitivity are thus poised to convey misleading information. In particular, if during an initial drug challenge the opposing responses that are recruited to counter the drug effect are unusually robust, the individual can appear insensitive to the drug owing to little apparent change in the measured variable. If the responses are lesser in magnitude and / or more sluggish to recruit, a change can nonetheless be observed in the measured variable and is often accompanied by the development of intrasessional or acute tolerance. Finally, if no effective opposing responses are made, a large change with minimal acute tolerance is observed in the measured variable. The essential point is that the appearance of initial insensitivity need not result from true biological insensitivity that renders a drug relatively inert, but rather that drug-elicited effects can be masked by opposing responses in highly responsive individuals [11, 13]. Indeed, describing initially insensitive individuals as having a “low level of response” during a drug challenge may, in some situations, be exactly the opposite of what is occurring. This insight was made possible because we were able to assess the mechanistic determinants of Tc (both HL and HP) in addition to Tc during the initial and final N_2_O challenge tests using continuous, non-invasive and sensitive methods.

The significance of high responsivity was particularly evident during the second N_2_O challenge. The II rats generated substantially more heat than was necessary to counter N_2_O’s hypothermic action, a response that caused the rats to become frankly *hyper*thermic [23, 24, 30]. This ‘sign-reversal,’ or overcompensation resulting from excessive effector activity, is not easily explained by common homeostatic interpretations and is instead characteristic of a dysregulated allostatic state [31]. Allostasis is considered to be metabolically inefficient and costly, resulting in allostatic load [32, 33]. Further, allostasis has been suggested to be pivotal in escalating drug-taking behavior [31, 34].

Recent investigations of N_2_O -induced allostasis indicate that not only do overcompensating effector responses drive the hyperthermic sign-reversal, but suggest that this overcompensation motivates cool-seeking behavior in a thermally-graded alleyway that opposes the over-compensated state [22, 35]. The present data suggest that an escalation of voluntary N_2_O inhalation might itself be a motivated behavior that is recruited to further increase HL (a primary drug effect), which counters the sign reversal caused by an allostatic HP response. However, increased drug consumption in these unusually responsive individuals provokes the development of a further increment in the autonomic HP counter-response that reinstates the sign reversal, favoring a further escalation of N_2_O inhalation to compensate for the effects of the excessive counter-response. This cycle of escalation can be viewed as an example wherein a complex biological control system that evolved a robust response to naturalistic thermoregulatory challenges is rendered fragile when faced with disturbances imposed by non-naturalistic challenges such as N_2_O inhalation. The present data are consistent with the possibility that high levels of initial reactivity and subsequent adaptability to N_2_O inhalation in the form of a HP response place the individual at greater risk for this fragility. Collectively, our findings provide empirical support for an allostatic model of drug addiction and suggest that a potential therapeutic strategy might be to target the hyper-reactive opposing responses that give the appearance of initial drug insensitivity and that eventually grow to become dysregulatory overactive compensatory responses.

The present study, as well as previous research [24], found that N_2_O’s effect to promote increased early heat loss persists across repeated administrations despite the development of chronic tolerance / allostasis at the level of Tc. Such a reliable effect on HL would always cause a loss of body temperature if not for a compensatory increase in HP. We have described the reliable increase in heat dissipation caused by N_2_O as a consequence of its pharmacological action while the more variable change of HP is thought to primarily reflect differential recruitment of response(s) responsible for the recovery and subsequent overcorrection of Tc during the development of tolerance / allostasis. This interpretation is based on a substantial body of research on the development of drug tolerance which indicates that opposing responses can offset a drug’s pharmacological effect during an initial drug administration (acute tolerance) and that as these opponent responses grow over repeated drug administrations, they more fully oppose the drug’s pharmacological effect and account for chronic drug tolerance [11, 36–38]. The experimental model [14] employed in the current study allows direct measurement of Tc and its underlying determinants, HP and HL, and the resulting findings are compatible with this view of tolerance development. The pattern of changes of HP over repeated drug exposures suggest that it reflects an opposing response that offsets the persistent drug effect of increased heat loss. A more complete discussion of the rationale for distinguishing N_2_O’s pharmacological effects from the co-occurring responses can be found in a previous article [13].

Changes in a broad spectrum of diverse measures (e.g., body sway, endocrine changes, self-report measures) assessed during a drug challenge test [9], as well as during other non-drug homeostatic challenges [39], have been found to reliably predict future drug taking. The current results expand the range of measures by demonstrating that an apparent initial insensitivity to a drug’s temperature-altering effects reflects an individual’s underlying pattern of physiological reactivity or responsiveness that is associated with greater subsequent drug self-administration. The diversity of measures that have been used to assess a drug’s initial impact on a dependent variable and subsequent drug taking suggests that the individual differences underlying addictive vulnerability may be broad and not limited to a single physiological system. An individual’s hyper-responsive phenotype may be a common vulnerability mechanism that impacts a variety of challenges to different physiological systems and that can be manifest as appearing initially insensitive in the case of a drug challenge. With continued drug exposure, excessive growth in the magnitude of opposing responses can lead to allostatic overcompensations that contribute to the development of addiction. Current models of allostasis also recognize the importance of stress responding (e.g., glucocorticoid secretion) and its effects on a broad range of physiological systems [31], and individual differences in responsiveness to stressors may be an important contributing factor to a hyper-responsive phenotype.

In a recent review, Piazza and Deroche-Gamonet [1] suggested that individuals vulnerable to developing drug addiction have both a phenotype that promotes the escalation of drug taking and sustained drug use, as well as a second phenotype that leads to loss of control over drug intake. “The necessity of having two distinct and independent vulnerable phenotypes to complete the transition to addiction explains why only a small number of individuals exposed to drugs develop the most severe form of the disease” (p. 398) [1]. These phenotypes can be assessed using behavioral challenges in preclinical research [40].

One well-studied example of such a phenotype is the high responder (HR) versus low responder (LR) model that has found that individual’s exhibiting a high locomotor response during a challenge test to a novel environment correlates with a greater secretion of glucocorticoids, an enhanced drug-induced release of dopamine in a brain reward center, and a subsequent increased tendency to escalate drug taking [41–45]. Piazza & DeRoche-Gamonet [46] emphasize that while the escalation of drug intake to sustained drug use is an inherent part of “any complete modeling of transition to addiction” (p. 3936) it is also important to assess whether an individual has lost control over drug taking to determine if an individual has become fully addicted (e.g., [1, 40, 47]). The present study investigated only the escalation of N_2_O self-administration and did not include measures to assess loss of control over drug taking behavior. Thus, it remains an open question whether individual differences in initial sensitivity during an initial drug challenge might predict both the escalation of drug taking as well as the loss of control components of a complete preclinical model of addiction.

In studies of individual differences, it is typical to classify subjects based on initial individual differences using a median split or upper versus lower quartiles with the more selective methods being more powerful because they accentuate the individual differences between the groups [48]. The present study used highly selective criteria to create the individual difference groups. Specifically, in each squad of rats that was screened for initial hypothermic sensitivity to N_2_O, only the two most sensitive and the two least sensitive rats were retained to form the individual difference groups. This selection strategy contributed to the clear findings that initially insensitive subjects are more likely to escalate their consumption of N_2_O when compared to initially sensitive individuals. Because the rats were selected from the entire sample, the findings indicate that rats selected using the same stringent criteria would exhibit a similar pattern of N_2_O self-administration. A reasonable hypothesis is that the non-selected subjects would exhibit an intermediate level of drug-taking behavior between these two individual difference groups in association with each individual’s degree of initial sensitivity, but this assumption remains untested.

If vulnerability to addiction is indeed based on an individual being hyper-responsive to challenges, it should be possible to elicit and thereby evaluate these response characteristics without the necessity of using a drug-challenge test. Future research should focus on developing non-drug challenges that identify individuals with relatively large effector responses, as this may lead to simpler and more practical ways of assessing a young individual’s addictive vulnerability.

## Supporting Information

S1 Fig Photograph of the N_2_O Self-Administration Apparatus. Photograph of the top two self-administration chambers of a four-shelf system. The water bottles that snap into the clip at the ends of each side chamber are displayed on the lower shelf.
(TIFF)

S2 Fig Self-Administration Data for Each II Rat. Self-administration data for each initially insensitive (II) rat are provided by dyad. A dyad consists of two 22-h data recording periods, which yields a total of 44 hours. The Y-axis is presented in hours with the maximum possible time during a dyad equal to 44 hours. The X-axis is presented in dyad number. The time spent in the central tub is indicated by the black dotted line; the time in the Control Gas side chamber is indicated by the red dashed line; and the time in the side chamber containing 60% N_2_O is indicated by the solid blue line. Missing data for dyads 4 and 5 (data collection error) for II rats: m and n.
(TIFF)

S3 Fig Self-Administration Data for Each IS Rat. Self-administration data for each initially sensitive (IS) rat are provided by dyad. A dyad consists of two 22-h data recording periods, which yields a total of 44 hours. The Y-axis is presented in hours with the maximum possible time during a dyad equal to 44 hours. The X-axis is presented in dyad number. The time spent in the central tub is indicated by the black dotted line; the time in the Control Gas side chamber is indicated by the red dashed line; and the time in the side chamber containing 60% N_2_O is indicated by the solid blue line. Missing data for dyads 4 and 5 (data collection error) for IS rats: L and H.
(TIFF)

S1 Dataset Data Underlying the Findings Presented in this Article. (XLSX)

S1 Movie Demonstration of the N_2_O Self-Administration Apparatus. A QuickTime movie clip demonstrates a rat using the self-administration apparatus.
(MP4)
