# Supplementary figures and images for: Predicting Addictive Vulnerability: Individual Differences in Initial Responding to a Drug’s Pharmacological Effects
Source: PLoS One. 2015 Apr 16;10(4):e0124740. doi: 10.1371/journal.pone.0124740 (PMC4400068; doi:10.1371/journal.pone.0124740)

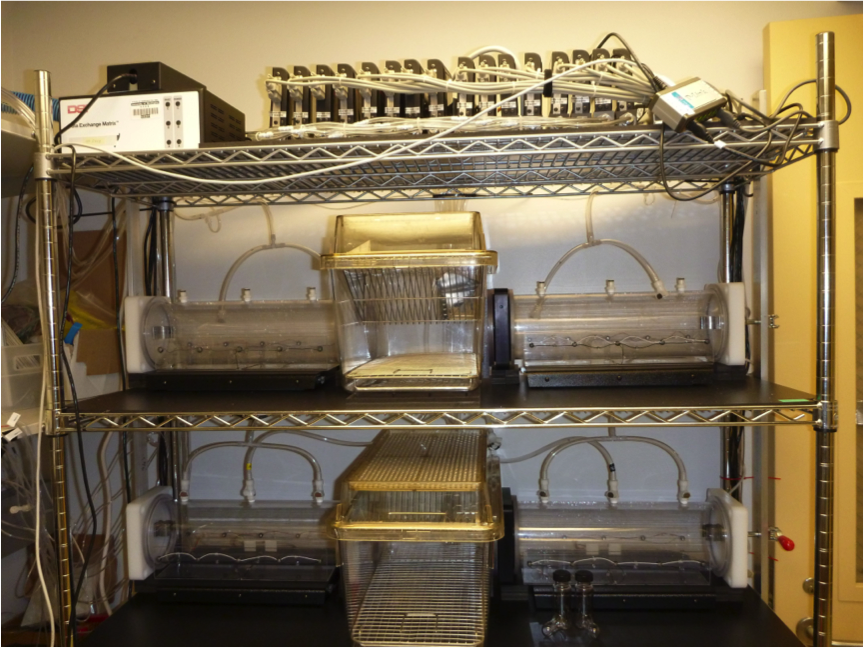

Supplement: S1 Fig — Photograph of the top two self-administration chambers of a four-shelf system. The water bottles that snap into the clip at the ends of each side chamber are displayed on the lower shelf. (TIFF) [file pone.0124740.s001.tiff]

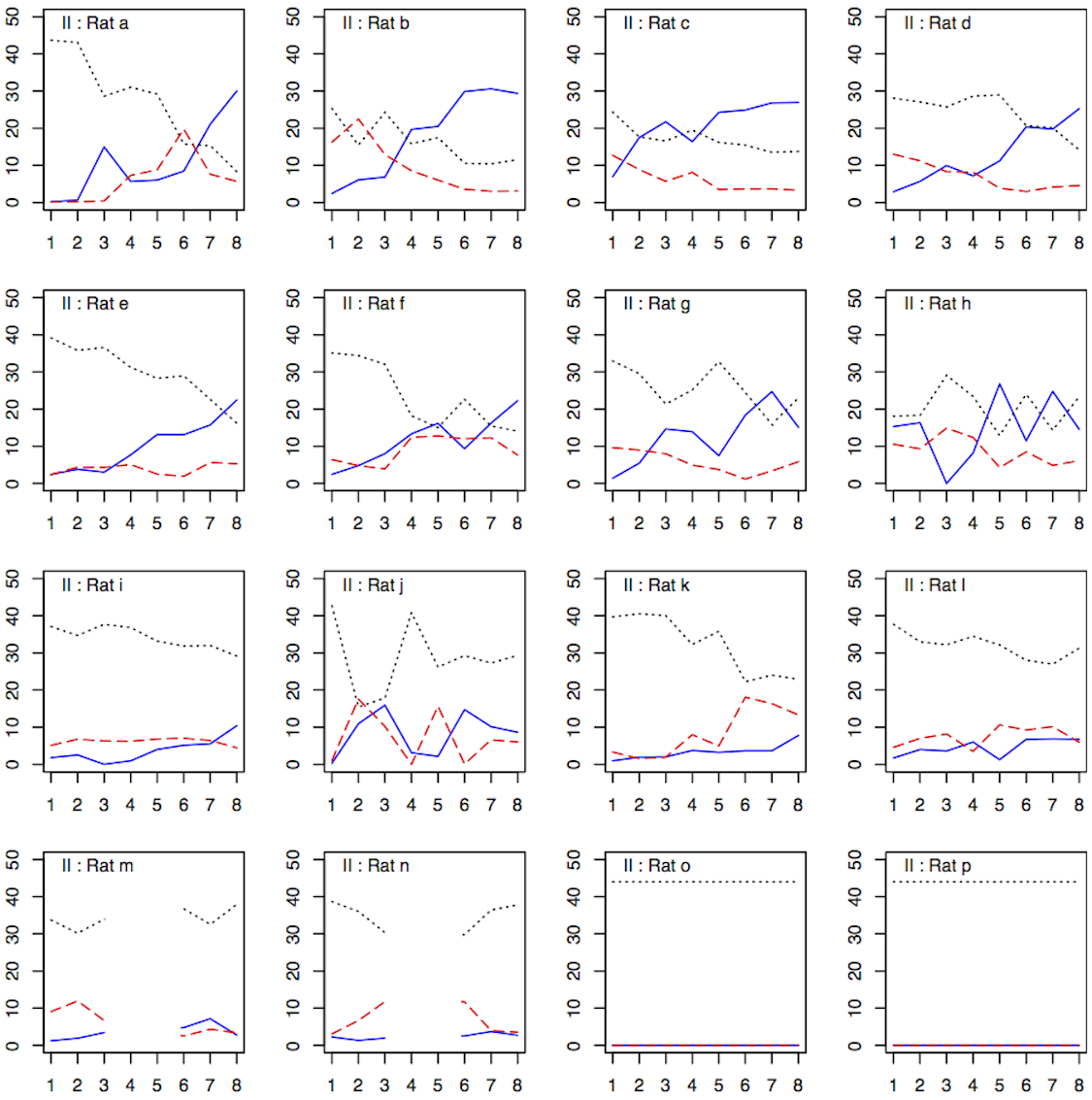

Supplement: S2 Fig — Self-administration data for each initially insensitive (II) rat are provided by dyad. A dyad consists of two 22-h data recording periods, which yields a total of 44 hours. The Y-axis is presented in hours with the maximum possible time during a dyad equal to 44 hours. The X-axis is presented in dyad number. The time spent in the central tub is indicated by the black dotted line; the time in the Control Gas side chamber is indicated by the red dashed line; and the time in the side chamber containing 60% N2O is indicated by the solid blue line. Missing data for dyads 4 and 5 (data collection error) for II rats: m and n. (TIFF) [file pone.0124740.s002.tiff]

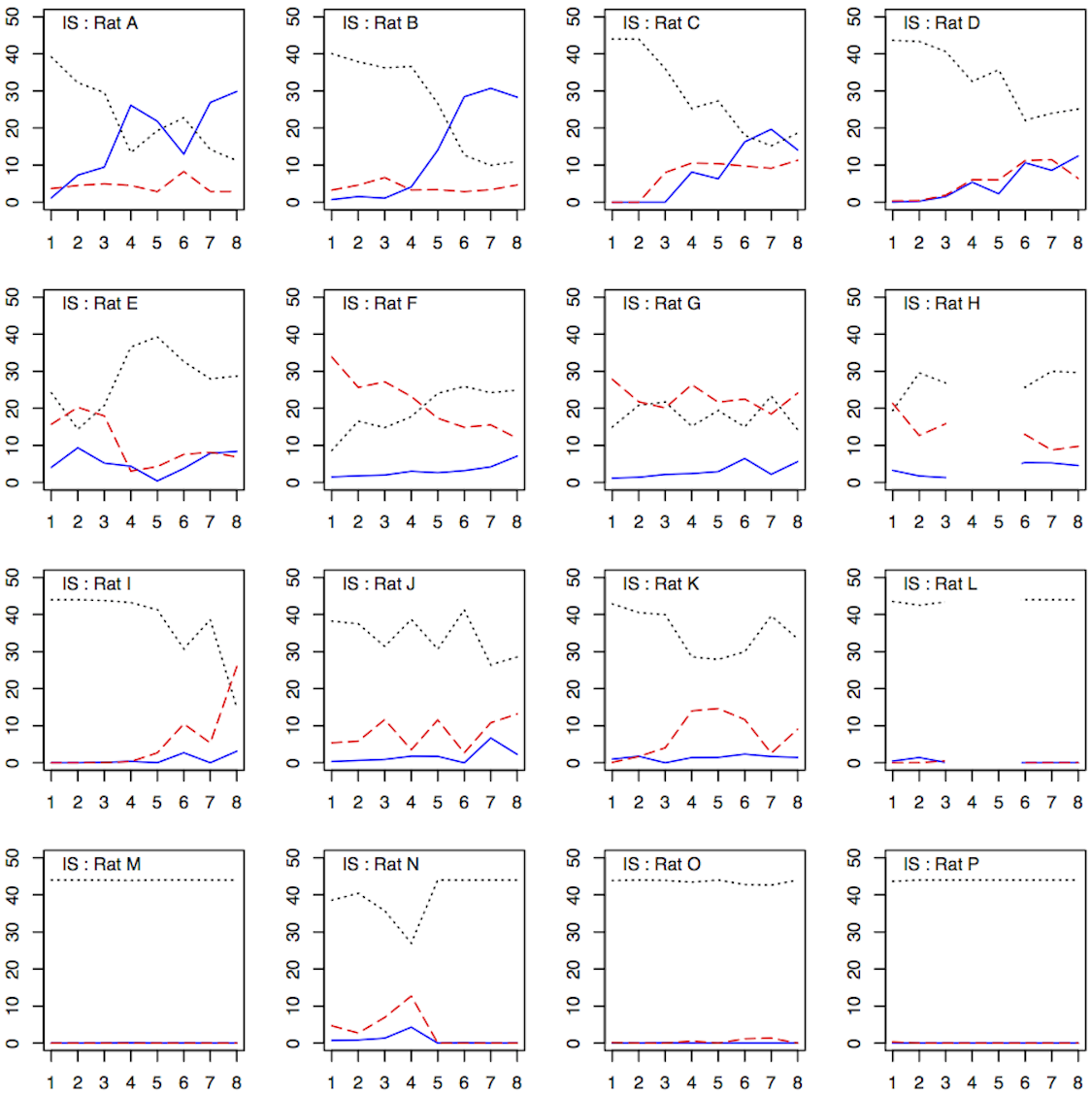

Supplement: S3 Fig — Self-administration data for each initially sensitive (IS) rat are provided by dyad. A dyad consists of two 22-h data recording periods, which yields a total of 44 hours. The Y-axis is presented in hours with the maximum possible time during a dyad equal to 44 hours. The X-axis is presented in dyad number. The time spent in the central tub is indicated by the black dotted line; the time in the Control Gas side chamber is indicated by the red dashed line; and the time in the side chamber containing 60% N2O is indicated by the solid blue line. Missing data for dyads 4 and 5 (data collection error) for IS rats: L and H. (TIFF) [file pone.0124740.s003.tiff]
